# *THRB* Gene Mosaicism Confirmed by Next-Generation Sequencing in a Clinically Symptomatic Infant

**DOI:** 10.1210/jcemcr/luae075

**Published:** 2024-05-03

**Authors:** Jenny Yeuk Ki Cheng, Shreenidhi Ranganatha Subramaniam, Hoi Shan Leung, Sammy Wai Chun Wong, Jeffrey Sung Shing Kwok, Wai Kei Jacky Lam

**Affiliations:** Department of Chemical Pathology, Prince of Wales Hospital, Hong Kong, China; Department of Chemical Pathology, The Chinese University of Hong Kong, Hong Kong, China; Department of Chemical Pathology, Prince of Wales Hospital, Hong Kong, China; Department of Chemical Pathology, The Chinese University of Hong Kong, Hong Kong, China; Department of Chemical Pathology, Prince of Wales Hospital, Hong Kong, China; Department of Department of Paediatrics & Adolescent Medicine, Alice Ho Miu Ling Nethersole Hospital, Hong Kong, China; Department of Chemical Pathology, Prince of Wales Hospital, Hong Kong, China; Department of Chemical Pathology, The Chinese University of Hong Kong, Hong Kong, China; Department of Chemical Pathology, Prince of Wales Hospital, Hong Kong, China; Department of Chemical Pathology, The Chinese University of Hong Kong, Hong Kong, China; Li Ka Shing Institute of Health Sciences, The Chinese University of Hong Kong, Hong Kong, China

**Keywords:** resistance to thyroid hormone β syndrome, *THRB*, mosaicism, next-generation sequencing

## Abstract

A 4-day-old infant was admitted for neonatal jaundice. He had persistent tachycardia and tachypnea. Initial workup showed a serum free T4 of 75.6 pmol/L (5.87 ng/dL) (reference range: 11.5-28.3 pmol/L; 0.89-2.20 ng/dL) and a nonsuppressed TSH 3.76 mIU/L (reference range: 0.72-11.0 mIU/L). A TRH stimulation test showed an exaggerated TSH response with a peak of 92.1 mIU/L at 30 minutes after TRH injection, which suggested the diagnosis of resistance to thyroid hormone β syndrome. Sanger sequencing showed a questionable pathogenic variant in the *THRB* gene with low signal amplitude. Restriction fragment length polymorphism was consistent with its presence. The variant was originally reported as heterozygous. Next-generation sequencing was performed on blood and buccal swab samples of the patient and his parents, which confirmed this de novo mosaic variant NM_000461.5:c.1352T > C p.(Phe451Ser) in the patient but not in his asymptomatic parents. As it was in a mosaic state, only the offspring, but not other first-degree relatives, of the patient would have the risk of inheriting that variant.

## Introduction

The *THRB* gene encodes 1 of the nuclear hormone receptors for T3. Resistance to thyroid hormone β syndrome (RTHβ) is a genetic condition that causes discordant thyroid function tests (TFTs). Patients usually have an elevated serum thyroid hormone (TH) with nonsuppressed serum TSH [1]. The proposed pathophysiology is related to the dominant negative effect of the mutant thyroid hormone receptor (THR) β protein on the wild-type THRs. The formation of dimers between mutant and wild-type THRβ can interfere with the function of wild-type THRβ, thereby inhibiting T3-mediated TH action [1]. Patients can manifest signs and symptoms of both hyperthyroidism and hypothyroidism, depending on the types of THRs expressed in the organs. For example, RTHβ patients usually present with sinus tachycardia because the heart predominantly expresses THRα. The high TH concentrations in RTHβ patients can bind to normally functioning THRα receptors, causing excessive hormonal actions at these sites. Meanwhile, the pituitary gland mainly expresses THRβ. The mutated THRβ in RTHβ resists the actions of TH, leading to the hypothyroid manifestations [1].

RTHβ is an autosomal dominant condition. Most cases (85%) carry a monoallelic pathogenic variant in the *THRB* gene [2]. Mosaic pathogenic variants, however, could be missed by Sanger sequencing due to low signal amplitude. Alternative sequencing methods like next-generation sequencing (NGS) could help confirm mosaicism. We present a case of a 4-day-old infant who had a clinical diagnosis of RTHβ, in whom a mosaic pathogenic variant in the *THRB* gene was identified, which was later confirmed by NGS.

## Case Presentation

A 4-day-old infant was admitted for neonatal jaundice in 2013. On admission, he had persistent tachypnea (more than 80-90 breaths per minute) and tachycardia (over 160 beats per minute). The electrocardiogram showed sinus tachycardia, while the echocardiogram was unremarkable. The patient was empirically put on antibiotics and was admitted to the intensive care unit for his persistent respiratory distress. He later developed high-output heart failure, which was controlled by diuretics.

## Diagnostic Assessment

The sepsis workup was unrevealing. Arterial blood gas, urea and electrolytes, and liver function tests, except bilirubin, were normal. Initial TFT showed a serum free T4 of 75.6 pmol/L (5.87 ng/dL) [reference range (RR): 11.5-28.3 pmol/L; 0.89-2.20 ng/dL] with nonsuppressed TSH 3.76 mIU/L (RR: 0.72-11.0 mIU/L). Serum free T3 was 17.8 pmol/L (11.6 pg/mL) (RR: 3.00-9.28 pmol/L; 1.95-6.04 pg/mL).

An alternative immunoassay platform yielded a similar TFT pattern, which made assay interference less likely. Antithyroid peroxidase and antithyroglobulin antibodies were negative. Serum α-subunit and SHBG were unremarkable. Ultrasonography of the thyroid gland showed mild enlargement without focal lesions. Differential diagnoses that remained were RTHβ and TSHoma. Therefore, a TRH stimulation test was performed. A less than 2-fold increase in TSH is expected in patients with TSHoma, while a more than 4-fold increase is expected in patients with RTHβ. The patient had an 18-fold increase in TSH, with a peak TSH of 92.1 mIU/L 30 minutes after TRH injection (baseline TSH 5.0 mIU/L). This favored the diagnosis of RTHβ.

**Figure 1.**
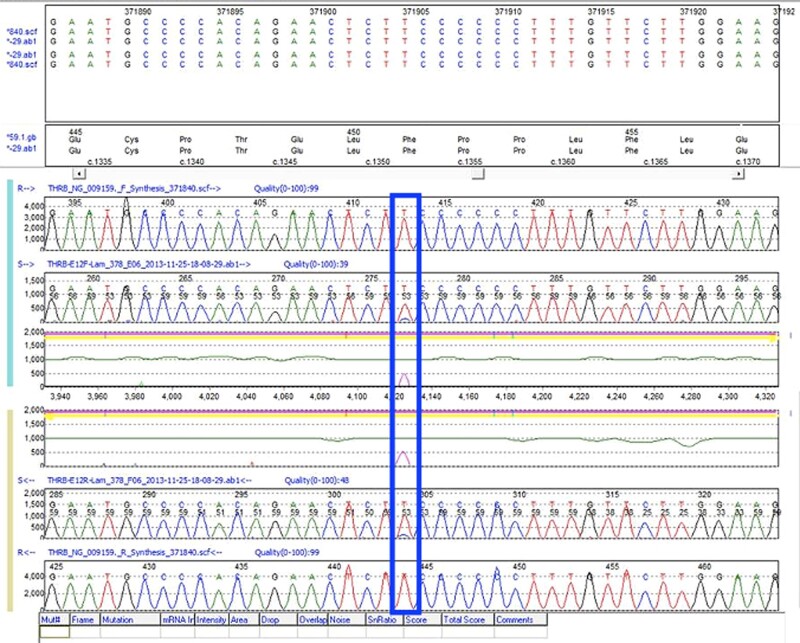
Sanger sequencing of the proband blood sample showed a questionable variant c.1352T > c at a low signal amplitude (variant highlighted in the box).

**Figure 2.**
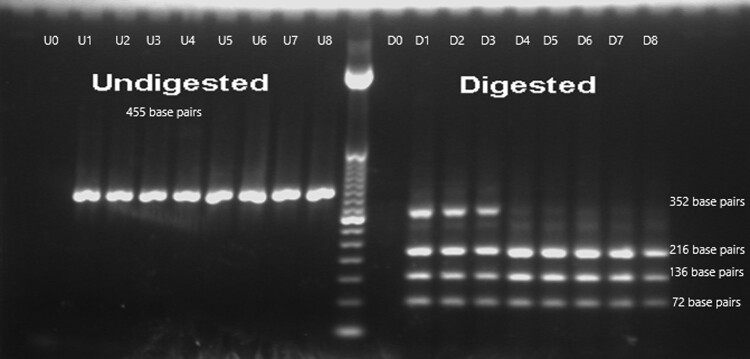
Gel image of restriction fragment length analysis. U0-U8: lanes of DNA without EarI enzymes, all with a size of 455 base pairs. U0: blank, U1-2: patient blood samples; U3: patient salivary sample; U4-U8: negative controls. D0-D8: lanes of DNA with EarI enzymes. D0: blank, D1-2: patient blood samples; D3: patient salivary sample; D4-D8: negative controls. For D1-3, 4 bands with sizes of 352, 216, 136, and 72 base pairs were observed, while for D4-8, only 3 bands with sizes of 216, 136, and 72 base pairs were observed.

Informed consent was obtained from the parents for genetic analysis of the patient. Sanger sequencing of exons 7 to 10 of the *THRB* gene (transcript NM_000461.5) in the patient's peripheral blood sample was performed. The polymerase chain reaction (PCR) conditions and primers are described in Table 1. A questionable variant c.1352T > C p.(Phe451Ser) was detected at a low signal amplitude (Fig. 1) and could not be called by Mutation Surveyor® DNA Variant Analysis Software (SoftGenetics, USA). It was reported as pathogenic in some publications [3, 4, 5]. Another peripheral blood sample as well as a salivary sample were analyzed by the same method and revealed the same results. Restriction fragment length polymorphism (RFLP) using EarI enzyme recognizing the sequence CTCTTC was performed on these samples, together with 5 negative controls. The same PCR conditions and primers for exon 10 were adopted. In the negative controls, only 3 bands with sizes of 216, 136, and 72 base pairs were observed, whereas an extra band of 352 base pairs was observed in the patient samples, which confirmed the presence of the target variant (Fig. 2). However, the variant allele frequency (VAF) could not be quantified accurately. Given the patient's clinical and biochemical findings, the variant was reported as heterozygous.

**Table 1.** Polymerase chain reaction conditions and primers for Sanger sequencing and next-generation sequencing

Methods	*THRB* (transcript NM_000461.5) exon	Primer sequence (5′ to 3′)	PCR conditions
Sanger sequencing	7	Forward: ATCAGTGGTCCCACTCCTG Reverse: CACCAGTATCCCAAGGTGATG	60 °C
	8	Forward: TCAGAAGAGATTTTCTGCCACA Reverse: TTCGTTTTGTACTGACGTTGC	58 °C
	9	Forward: GAAAACCATGGGCTCAAAGA Reverse: AGCGCTAGACAAGCAAAAGC	56 °C
	10	Forward: TAAAGGCCTGGAATTGGACA Reverse: TCCCTCCCAACACAAAGAAA	60 °C
Next-generation sequencing	7	Forward: AGAGCTAGGCAATGGAATGAAATGACAC Reverse: CTGGAATTGGACAAAGCAAGCCTTC	66 °C
	8	Forward: CTGAAGAAGAGTGAGCTATGTTTCTGAAGC Reverse: TGACATGAACTGGTTCTTTTCAGCTGC	66 °C
	9	Forward: CCTCACCTCACAAAACATAGA/GCAACT Reverse: GCAGCAACAGTCCTGTAAACATTGTCA	66 °C
	10	Forward: CTTTGCAAGTTACTCAGCCTCTCAGA Reverse: CATCTGTGTGCCTTGTCTCATCTTTCTC	66 °C

Abbreviations: PCR, polymerase chain reaction.

## Treatment

The patient was initially put on propylthiouracil 4 mg thrice daily and propranolol 0.57 mg/kg/day for heart rate control. However, the use of propylthiouracil increased the serum TSH to 97.5 mIU/L (reference range: 0.72-11.0 mIU/L) and was thus weaned off. The patient was discharged after 2 months, with a serum TSH of 6.77 mIU/L (reference range: 0.72-11.0 mIU/L) and serum free T4 of 69.6 pmol/L (5.40 ng/dL) (reference range: 11.5-28.3 pmol/L; 0.89-2.20 ng/dL) before discharge.

## Outcome and Follow-up

The patient has been followed up at the pediatric clinic. Propranolol was titrated up to 0.88 mg/kg/day, but the resting heart rate remained high at 140 to 150 beats per minute. Therefore, when he was 10 months old, propranolol was switched to atenolol, a long-acting cardioselective β-antagonist, with a starting dose of 1.3 mg/kg/day. The heart rate control was satisfactory when atenolol was titrated up to 2 mg/kg/day. Regular monitoring by echocardiography was unremarkable.

His bone age was significantly advanced compared to his chronological age. He was noted to have developmental delay and borderline intellectual abilities when he was 3 years old. He was inattentive at school and had to repeat kindergarten one. 35,3-triiodothyroacetic acid (TRIAC) was then started, after which the patient showed improvements in his bone age and hyperactivity symptoms. The local supply of TRIAC was, however, in shortage when he was around 6 years old, and his inattention and hyperactivity symptoms worsened after he completely stopped the medication. He was later diagnosed with attention deficit hyperactivity disorder and treated with methylphenidate, with an initial dose of 5 mg twice daily. His condition improved, but he still had poor academic performance. His current regimen includes atenolol 50 mg daily and methylphenidate 30 mg twice daily. Biochemical and clinical features are summarized in Table 2.

**Table 2.** Clinical and biochemical progress of the patient

Date	Actual age	Bone age	Serum free T4 (RR: 11.5-28.3 pmol/L; 0.89-2.20 ng/dL)	Serum TSH (RR: 0.72-11.0 mIU/L)	Remarks
Jun 2014	8 months	/	38.5 pmol/L (2.99 ng/dL)	1.8 mIU/L	Propranolol was switched to atenolol
Dec 2014	1 years and 2 months	32 months	46.8 pmol/L (3.64 ng/dL)	4.48 mIU/L	
Nov 2015	2 years and 1 month	60 months	56.7 pmol/L (4.40 ng/dL)	3.3 mIU/L	
May 2017	3 years and 7 months	/	52.1 pmol/L (4.05 ng/dL)	4.7 mIU/L	TRIAC was started since Jun 2017
Nov 2017	4 years and 1 month	6 years	38.9 pmol/L (3.02 ng/dL)	4.87 mIU/L	
Dec 2018	5 years and 2 months	7 years	47.8 pmol/L (3.71 ng/dL)	3.78 mIU/L	
Jul 2019	5 years and 9 months	9 years	41.1 pmol/L (3.19 ng/dL)	4.25 mIU/L	TRIAC was stopped since Sept 2019
Oct 2019	6 years	/	58.9 pmol/L (4.58 ng/dL)	3.24 mIU/L	
Mar 2020	6 years and 5 months	11 years	40.2 pmol/L (3.12 ng/dL)	7.93 mIU/L	TRIAC was restarted since Mar 2020
Jul 2020	6 years and 9 months	/	31.2 pmol/L (2.42 ng/dL)	2.48 mIU/L	TRIAC was stopped since Oct 2020
Jan 2021	7 years and 3 months	/	/	/	Patient was diagnosed with attention deficit hyperactivity disorder
Oct 2021	8 years	13 years	42 pmol/L (3.26 ng/dL)	2.66 mIU/L	
Dec 2022	9 years and 2 months	13.18 years	37.4 pmol/L (2.91 ng/dL)	3.5 mIU/L	

Values in parentheses are conventional units.

Abbreviations: RR, reference range; TRIAC, 35,3-triiodothyroacetic acid.

A high-throughput NGS analyzer has been available in our laboratory since 2022. Therefore, this case was reanalyzed with NGS to confirm the mosaicism. Multiplexed PCR, targeting exons 7 to 10 of the *THRB* gene (Table 1), was performed in the patient's peripheral blood sample, together with 10 negative controls. The amplified DNA products then underwent deep sequencing. The same c.1352T > C variant was detected only in the patient's sample, with a VAF of 16.80% in almost 8 million reads. Subsequently, another peripheral blood, urine, and buccal swab sample were obtained from the patient and analyzed with the same approach. Peripheral blood leukocytes and buccal swab samples detected the same variant, with a VAF of 24.64% (in around 2 million reads) and 19.87% (in around 1 million reads), respectively. The urine sample was not analyzed due to low DNA content. The c.1352T > C variant was not detected in the blood, urine, or buccal swab samples of the asymptomatic parents. The state of the variant in the proband was finally updated from “heterozygous” to “mosaic.”

## Discussion

In this report, we presented a case of clinical RTHβ having a mosaic pathogenic *THRB* variant confirmed by NGS.

RTHβ is the most common genetic cause of discordant TFTs. The typical biochemical picture is elevated TH with nonsuppressed TSH. The clinical picture includes thyroid-related features like tachycardia and goiter, as well as neurological features like learning disabilities, hyperactivity, and developmental delay, as illustrated in our patient. Yet some patients can be completely asymptomatic [6].

There are more than 150 disease-causing mutations of the *THRB* gene in the Human Gene Mutation Database. For the transcript NM_000461.5, known disease-causing variants accounting for RTHβ occur over exons 7 to 10. The variant NM_000461.5:c.1352T > C p.(Phe451Ser) was reported as disease-causing in the Human Gene Mutation Database 2023.4 and identified in patients with RTHβ [3, 4, 5]. It is in exon 10, which is considered a mutational hotspot [7]. Multiple in silico analyses also suggest its pathogenicity. Mutations affecting the same codon, namely p.(Phe451Ile) and p.(Phe451Leu), have been reported as pathogenic [8, 9]. It is a de novo variant only present in the patient but not in his asymptomatic parents. Therefore, this variant is classified as pathogenic according to the American College of Medical Genetics and Genomics and the Association for Molecular Pathology guidelines [10].

Mosaicism is defined by the presence of 2 genetically distinct populations of cells with different population sizes. It is caused by postzygotic spontaneous mutations that occur during early embryonic or fetal development [11]. As shown in our case, the parents of the proband do not carry the pathogenic variant. Indeed, the parents of a patient with mosaic pathogenic variant(s) have no increased risk of having another affected child. Nevertheless, the patient may pass the variant(s) to his/her offspring if the mosaicism occurs in the gonads, and the mosaic variant will become a germline heterozygous variant in the offspring [11]. Therefore, unlike patients with heterozygous variant(s), only the offspring, but not all first-degree relatives, of probands with mosaic variant(s) are at risk of inheriting the variant(s) and should receive genetic counseling and/or cascade screening.

There are 2 published case reports about *THRB* mosaicism [12, 13]. The case reported by Donnars et al was a patient clinically diagnosed with RTHβ who had a negative genetic test by Sanger sequencing. NGS, however, identified a mosaic pathogenic variant c.949G > A p.(Ala317Thr) with VAFs ranging from 0% to 17.9% in peripheral blood leukocytes, conjunctival and oral epithelium. The case from Mamanasiri et al was an asymptomatic father of 2 RTHβ children. He was found to have discordant TFTs during cascade screening. RFLP detected a mosaic pathogenic variant c.1012C > T p.(Arg338Trp) in his peripheral blood leukocytes (VAF 8.6%) and prostate (VAF 3.9%). These case reports demonstrated that patients with *THRB* mosaicism could have a wide variety of presentations and different VAFs in different tissues, and the mosaicism could be detected by different sequencing methods.

Around 15% of RTHβ patients have negative genetic tests [2]. However, some of those “wild-type” patients may actually carry mosaic pathogenic variant(s) that have been missed by Sanger sequencing due to low signal amplitude. It might therefore be beneficial to use alternative sequencing approaches for those Sanger-negative cases. Although RFLP could help identify suspected mosaicism, it requires the use of specific restriction enzymes and cannot quantify the VAF accurately. Nowadays, as NGS becomes more readily available in most genetic laboratories, it would be the method of choice for detecting mosaicism due to its high detection sensitivity and high throughput [14].

In summary, we reported a case of RTHβ with a mosaic pathogenic variant in the *THRB* gene as confirmed by NGS. Identifying mosaic variants facilitates the clinical management and genetic counseling of patients, as this implies only the offspring but not other first-degree relatives should receive cascade screening. It is thus worthwhile to perform NGS when there is suspicion of mosaicism, especially in Sanger-negative cases. Samples from different tissue origins should be obtained to increase the sensitivity.

## Learning Points

- Resistance to thyroid hormone β syndrome is an autosomal dominant condition. However, 15% of patients have no pathogenic variant in the THRB gene.
- The most common sequencing technique for the THRB gene is Sanger sequencing. Yet this can miss the symptomatic patients with mosaic pathogenic variants due to low signal amplitude.
- Next-generation sequencing for those Sanger-negative or Sanger-ambiguous cases would be useful to detect mosaic variants and quantify the variant allele frequency.
- Correctly identifying symptomatic patients with the THRB mosaic variant is important as this affects the clinical management and genetic counseling. Unlike cases with heterozygous variants, those patients will only potentially pass the variant to their offspring. All other first-degree relatives are unaffected.

## Data Availability

Some or all datasets generated during and/or analyzed during the current study are not publicly available but are available from the corresponding author on reasonable request.
